# Mr. John Evans

**Published:** 1911-08-26

**Authors:** 


### OBITUARY.

The death is announced of Mr. John Evans, M.R.C.S. Eng., L.D.S. He had retired from practice for some time. He was one of the earliest students of Charing Cross Hospital, and became a member of the Royal College of Surgeons in 1857, and took the licentiate in dental surgery in 1860.

